# Early detection and surveillance of SARS-CoV-2 genomic variants in wastewater using COJAC

**DOI:** 10.1038/s41564-022-01185-x

**Published:** 2022-07-18

**Authors:** Katharina Jahn, David Dreifuss, Ivan Topolsky, Anina Kull, Pravin Ganesanandamoorthy, Xavier Fernandez-Cassi, Carola Bänziger, Alexander J. Devaux, Elyse Stachler, Lea Caduff, Federica Cariti, Alex Tuñas Corzón, Lara Fuhrmann, Chaoran Chen, Kim Philipp Jablonski, Sarah Nadeau, Mirjam Feldkamp, Christian Beisel, Catharine Aquino, Tanja Stadler, Christoph Ort, Tamar Kohn, Timothy R. Julian, Niko Beerenwinkel

**Affiliations:** 1 Department of Biosystems Science and Engineering, ETH Zurich, Basel, Switzerland; 2 SIB Swiss Institute of Bioinformatics, Lausanne, Switzerland; 3 Eawag, Swiss Federal Institute of Aquatic Science and Technology, Dübendorf, Switzerland; 4 Laboratory of Environmental Chemistry, School of Architecture, Civil and Environmental Engineering, École Polytechnique Fédérale de Lausanne (EPFL), Lausanne, Switzerland; 5 Functional Genomics Center Zurich, ETH Zurich, Zurich, Switzerland; 6 Swiss Tropical and Public Health Institute, Basel, Switzerland; 7 University of Basel, Basel, Switzerland

**Keywords:** Statistical methods, Viral infection, Software

## Abstract

The continuing emergence of SARS-CoV-2 variants of concern and variants of interest emphasizes the need for early detection and epidemiological surveillance of novel variants. We used genomic sequencing of 122 wastewater samples from three locations in Switzerland to monitor the local spread of B.1.1.7 (Alpha), B.1.351 (Beta) and P.1 (Gamma) variants of SARS-CoV-2 at a population level. We devised a bioinformatics method named COJAC (Co-Occurrence adJusted Analysis and Calling) that uses read pairs carrying multiple variant-specific signature mutations as a robust indicator of low-frequency variants. Application of COJAC revealed that a local outbreak of the Alpha variant in two Swiss cities was observable in wastewater up to 13 d before being first reported in clinical samples. We further confirmed the ability of COJAC to detect emerging variants early for the Delta variant by analysing an additional 1,339 wastewater samples. While sequencing data of single wastewater samples provide limited precision for the quantification of relative prevalence of a variant, we show that replicate and close-meshed longitudinal sequencing allow for robust estimation not only of the local prevalence but also of the transmission fitness advantage of any variant. We conclude that genomic sequencing and our computational analysis can provide population-level estimates of prevalence and fitness of emerging variants from wastewater samples earlier and on the basis of substantially fewer samples than from clinical samples. Our framework is being routinely used in large national projects in Switzerland and the UK.

## Abstract (web summary)

The bioinformatics method COJAC enables improved population-level surveillance of the emergence and spread of SARS-CoV-2 variants in wastewater.

## Main

The ongoing spread and evolution of SARS-CoV-2 has generated several variants of interest and variants of concern (VOC)^1–3^, which can affect, to different degrees, transmissibility^1^, disease severity^4^, diagnostics and the effectiveness of treatment^5^ and vaccines. Therefore, early detection and monitoring of local variant spread has become an important public health task^6^.

Viral RNA of SARS-CoV-2 infected persons can be detected in the sewage collected in wastewater treatment plants (WWTPs) and its concentration has been shown to correlate with case reports^7^. Moreover, wastewater samples can provide a snapshot of the circulating viral lineages and their diversity in the community through reverse transcription quantitative real-time PCR (RT-qPCR) analysis^8,9^ or genomic sequencing^9–16^. Recently, it has been shown that variant prevalence in wastewater correlates with clinical data^17,18^. Therefore variant monitoring in wastewater may serve as an efficient and complementary approach to genomic epidemiology based on individual patient samples.

However, it is challenging to analyse wastewater samples for their SARS-CoV-2 genomic composition because concentrations of SARS-CoV-2 can be very low, samples may be enriched for PCR inhibitors, viral genomes are typically fragmented and sewage contains large amounts of bacterial, human and other viral DNA and RNA genomes. In addition, the quality of the data obtained from sequencing the mixture of viral genomes is compromised by amplification biases, sequencing errors and incomplete phasing information, which further complicates the detection of an emerging viral lineage that is present in only a small fraction of infected persons.

**Fig. 1:**
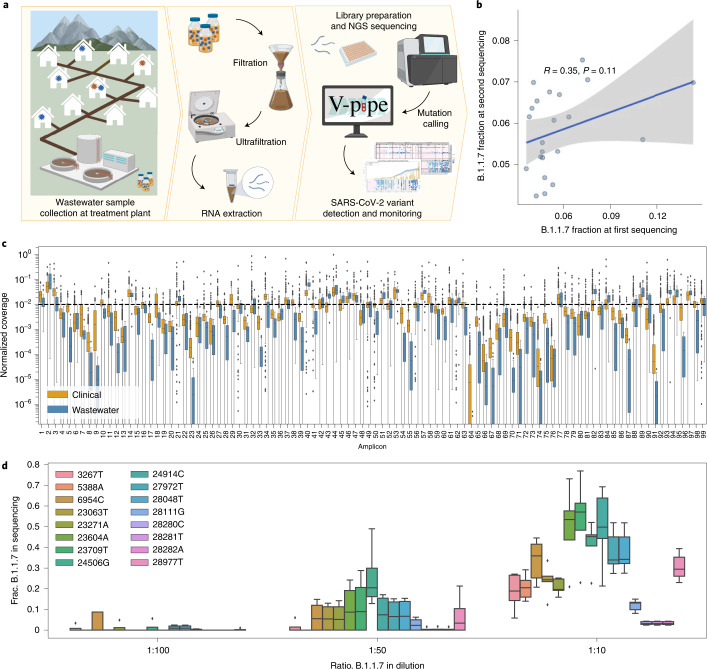
Method overview and quality control. **a**, Overview of the wastewater sampling campaign. Left: collection of raw wastewater samples containing a mixture of wild-type and variant SARS-CoV-2 viral RNA. Middle: viral concentration and nucleic acid extraction. Right: amplification using ARTIC v3 primers, library preparation, NGS and mutation calling using V-pipe, followed by statistical analysis to detect and quantify the presence of SARS-CoV-2 variants and estimate epidemiological parameters. Created with BioRender.com. **b**, Reproducibility of Alpha (B.1.1.7) prevalence based on resequencing of 25 samples. Each dot shows the average fraction of Alpha-compatible reads across all signature mutations. Pearson correlation coefficient, *R*, and *P* value (two-sided test) indicate a high degree of variability in Alpha prevalence estimates at low frequencies. The solid line denotes the estimate from the linear model and the shaded area denotes the 95% confidence interval. **c**, Per-amplicon normalized coverage distributions after quality filtering and alignment in the same NGS batch containing both 589 clinical (orange) and 22 wastewater (blue) samples. Per-amplicon absolute coverages can be found in Supplementary Fig. 1. **d**, Reproducibility of Alpha (B.1.1.7) prevalence in a dilution series experiment. Boxplots represent fractions of substitutions called in 5 technical replicates of wastewater spiked with SARS-CoV-2 RNA at 3 different Alpha-to-wild-type ratios. In both **c** and **d**, boxes show quartiles and the whiskers extend to a maximum of 1.5× the interquartile range, after which points are considered outliers. Source data

Here we analysed amplicon-based next-generation sequencing (NGS) data of viral RNA extracted from raw influent samples obtained from multiple Swiss WWTPs (Fig. 1a). To assess reproducibility and quantifiability of sequencing data obtained from wastewater-derived viral RNA, we conducted a series of replicate and spike-in experiments. We then focussed on a close-meshed time-series in two large cities between December 2020 and mid-February 2021, and a ski resort during the holiday season (121 samples in total). These samples cover the period in which the Alpha, Beta and Gamma variants first arrived in Europe. For validation, we then analysed 1,656 mostly daily samples from six WWTPs taken between January and September 2021 to cover the period in which the delta variant emerged. We developed a bioinformatics method named COJAC (Co-Occurrence adJusted Analysis and Calling) for early detection of low-frequency variants emerging in a population and a statistical approach that is suitable for quantitative variant monitoring and estimation of the variant-specific transmission fitness advantage (that is, the relative increase in reproductive number) of any genetic variant of SARS-CoV-2. Our framework works best on close-meshed time-series data.

## Results

### Quality of genomic sequencing data derived from wastewater samples

To assess the quality of genomic sequencing data derived from wastewater samples, we compared it to clinical sequencing data. We found that the normalized amplicon coverage obtained from the wastewater samples was not substantially different from the coverage of clinical samples (Fig. 1c) and that it allowed for calling low-frequency mutations in most genomic regions of most wastewater samples we analysed (Supplementary Fig. 1). Replicate and spike-in experiments (Methods) indicate that the relative prevalence of genomic variants can be quantified from the NGS data, although precision is limited at low prevalence. Replication increases precision, especially in the monitoring of low-frequency variants (Fig. 1b,d and Supplementary Information).

### Longitudinal surveillance of the Alpha, Beta and Gamma variant

**Fig. 2:**
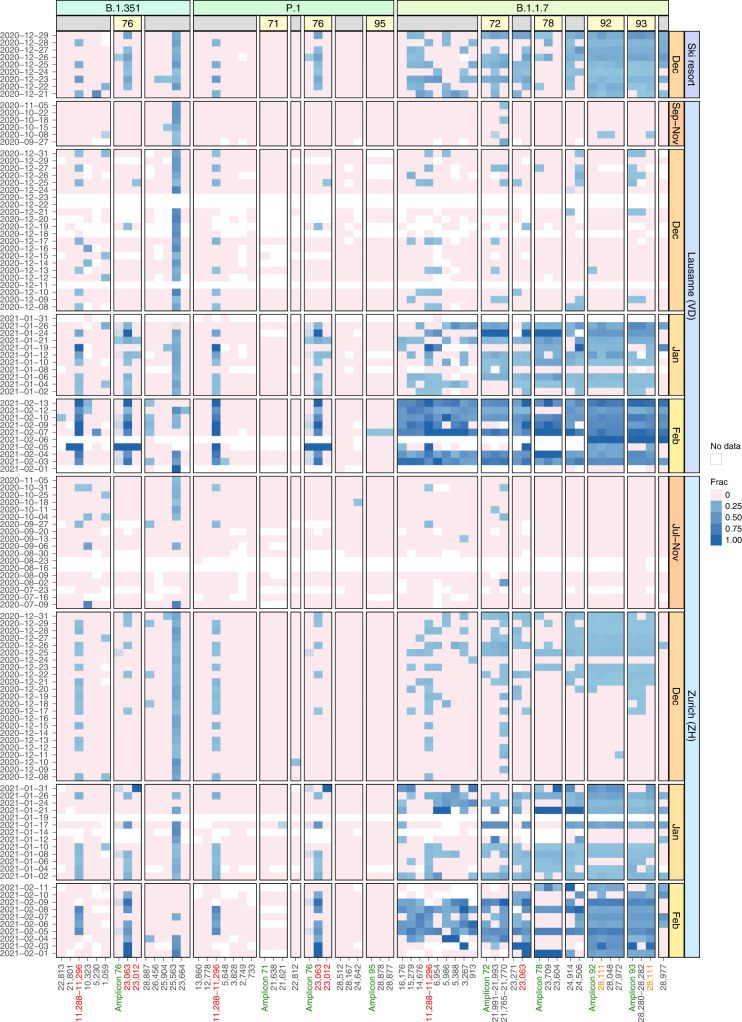
Longitudinal surveillance of Alpha (B.1.1.7), Beta (B.1.351) and Gamma (P.1) signature mutations in wastewater samples collected at three Swiss WWTPs. Blue color shading encodes the observed fraction of each signature mutation in each sample, pink indicates absence of the mutation and white indicates missing values (due to insufficient coverage). Mutations are grouped by variant and further by amplicon number (yellow boxes) in case multiple mutations co-occur on the same amplicon. Columns labelled ‘Amplicon’ followed by a number (green) show the observed frequency of co-occurrence on the same read pair for all mutations located on the respective amplicon. Mutations occur multiple times on the *y* axis if they either occur in more than one variant (red) or are located on two overlapping amplicons (orange). Source data

**Fig. 3:**
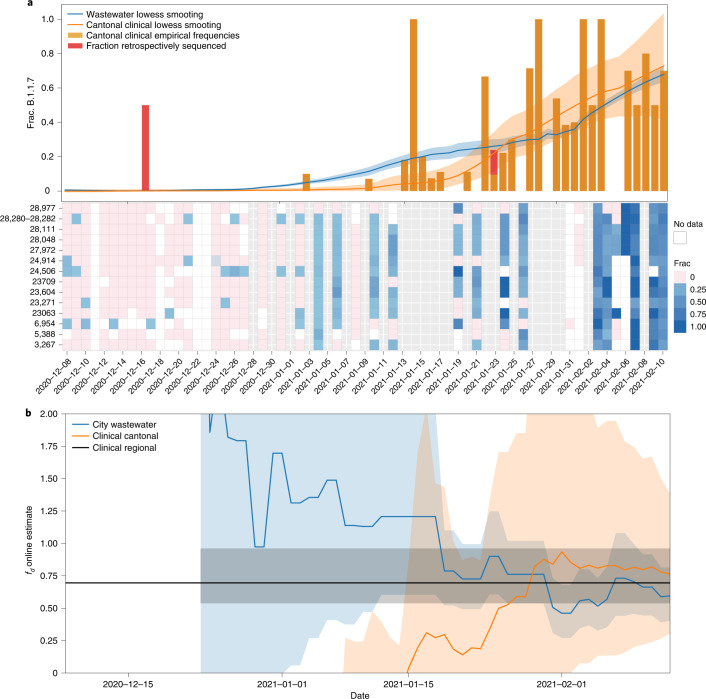
Prevalence and fitness advantage estimation for Lausanne based on wastewater and clinical sequencing data. **a**, Top: Alpha (B.1.1.7) prevalence estimates based on wastewater sequencing data and on cantonal clinical sequencing data for Lausanne. Bottom: frequencies of Alpha-characteristic substitutions found in wastewater sequencing samples, which are aggregated and smoothed in the top panel. Grey columns show dates without wastewater samples. White columns show dates of failed experiments (insufficient coverage/no SARS-CoV-2 RNA detected in the sample). Orange and red bars indicate the frequency of Alpha-positive cantonal clinical samples, which are also smoothed. The red parts indicate the fraction of Alpha-positive samples that were sequenced retrospectively in March/April 2021 (cut-off date for the GISAID submission date, 21 March 2021). Solid lines represent the smoothed estimates and shaded areas represent 95% confidence bands. **b**, Estimates of the transmission fitness advantage *f*_d_, computed online (Methods) using the wastewater (blue) and cantonal clinical (orange) sequencing data only until the respective timepoints. Solid lines represent the maximum likelihood estimates, shaded areas represent 95% confidence intervals and the horizontal black line indicates offline estimate of *f*_d_ based on clinical samples of the Lake Geneva Region dated 14 December 2020 to 11 February 2021 from Chen et al.^19^. Source data

**Fig. 4:**
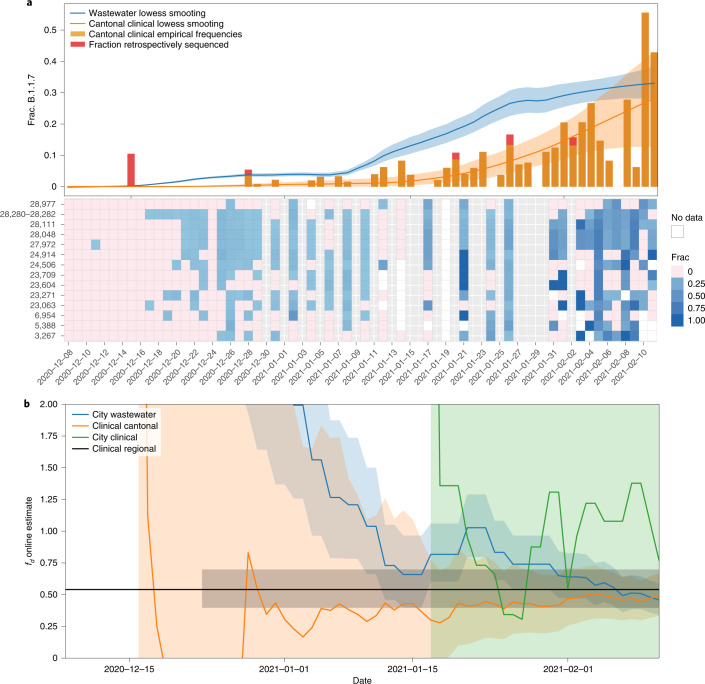
Prevalence and fitness advantage estimation for Zurich based on wastewater and clinical sequencing data. **a**, Top: Alpha (B.1.1.7) prevalence estimates based on wastewater sequencing data and on cantonal clinical sequencing data for Zurich. Bottom: frequencies of Alpha-characteristic substitutions found in wastewater sequencing samples, which are aggregated and smoothed in the top panel. Grey columns show dates without wastewater samples. White columns show dates of failed experiments (insufficient coverage/no SARS-CoV-2 RNA detected in the sample). Orange and red bars indicate the frequency of Alpha-positive cantonal clinical samples, which are also smoothed. The red parts indicate the fraction of Alpha-positive samples that were sequenced retrospectively in March/April 2021 (cut-off date for the GISAID submission date, 21 March 2021). Solid lines represent the smoothed estimates and shaded areas represent 95% confidence bands. **b**, Estimates of the transmission fitness advantage *f*_d_, computed online (Methods) using the wastewater (blue), cantonal clinical (orange) and city clinical (green) sequencing data only until the respective timepoints for Zurich. Solid lines represent the maximum likelihood estimates, shaded areas represent 95% confidence intervals and the horizontal black line indicates offline estimate of *f*_d_ based on clinical samples of the Greater Zurich Area dated 14 December 2020 to 11 February 2021 from Chen et al.^19^. Source data

The variant frequencies in the 122 wastewater samples revealed a continuous increase in the prevalence of the Alpha variant in Zurich starting around mid-December and in Lausanne starting in late December (Fig. 2). Much of the noise in the data can be removed by computing smoothed estimates over time and over signature mutations (Methods). When comparing these estimates of Alpha prevalence to those obtained from clinical samples, we found that they aligned very closely, even though the treatment plants serve only a subset of the respective cantonal populations (Lausanne: 30% of canton Vaud, Zurich: 29% of canton Zurich) (Figs. 3 and 4). For the alpine ski resort, we detected the Alpha variant over the entire period of observation (20–29 December 2020), consistent with the popularity of the ski resort with British tourists as a holiday destination (Fig. 2). Unlike for Alpha, we found almost no evidence for the distinctive signature mutations of Beta or Gamma (Supplementary Information), which is consistent with the observation that neither of the two variants was able to establish itself in the Swiss population.

### Early detection of emerging variants with COJAC

For early detection and determination of the timing of the introduction of a variant into a population, we devised the bioinformatics method COJAC that searches for co-occurrence of mutations on read pairs (Methods). Such co-occurrence signals provide high confidence in the presence of the respective strain, as independent biological generation or technical artefacts are both very unlikely to produce such mutational patterns (Supplementary Information). We analysed all amplicons that contained co-occurrences of VOC-specific mutations: four amplicons that each contain two or three Alpha-defining mutations, one amplicon with two signature mutations shared between Beta and Gamma, and one amplicon with two Gamma signature mutations. We found several co-occurrences in our data (Fig. 2 and Supplementary Table 3). The two mutations co-located on amplicon 93 provide the earliest evidence for Alpha in wastewater samples in Zurich on 17 December and in Lausanne on 9 December. In both locations, these dates fall at a time when evidence based on single mutations alone was still very spotty (Fig. 2).

We compared our results to early variant detection based on clinical sequencing data in the respective cantons of Zurich and Vaud. In Switzerland, around 4% of all SARS-CoV-2-positive clinical samples of December 2020 were sequenced. The first clinical evidence of the Alpha variant in canton Zurich was detected in a sample dated 18 December, and in canton Vaud in a sample dated 21 December, the former being 1 d later and the latter being 13 d later than the first wastewater-based evidence. This finding is consistent with those of a retrospective sequencing campaign of March/April 2021 analysing clinical samples collected in November and December 2020. The retrospectively obtained data revealed isolated occurrences of Alpha already on 9 November (3 cases) in canton Zurich, and on 17 December in canton Vaud (1 case) (Supplementary Table 5). For canton Vaud, the retrospective variant detection was still significantly later (8 d) than the first wastewater-based evidence in its capital Lausanne. For canton Zurich, the Alpha-positive samples of 9 November all originated from municipalities located outside the catchment area of the studied treatment plant and therefore could not be detected in the analysed wastewater samples.

The co-occurring mutation pair on amplicon 93 that we used for early detection in wastewater is highly specific to Alpha, as it has been observed only 14 times (0.07%) outside of the Alpha lineage in the 21,163 Swiss samples in the GISAID database (Supplementary Table 4) and only 138 times (0.01%) in all 1,397,333 SARS-CoV-2 GISAID samples until 13 February 2021. At later timepoints, we also observe evidence based on the other three Alpha-specific amplicons both in Lausanne and Zurich. In the ski resort, evidence for the presence of Alpha has already been strong based on the analysis of individual mutations over the entire period of observation (20–29 December), and the amplicon-based analysis further supports this observation. For Beta and Gamma, the evidence based on co-occurrence is similarly weak as for the analysis of individual mutations (Supplementary Information). This result aligns with the clinical data for the period of study, with only one Gamma sample detected in canton Zurich (first occurrence on 27 January) and none in canton Vaud. Beta was detected two times in canton Zurich (first occurrence 18 January) and seven times in canton Vaud (first occurrence 14 January).

### Estimation of transmission fitness advantage

The transmission fitness advantage of a variant corresponds to the relative increase in reproductive number and provides information on the epidemiological relevance of an emerging variant. We estimated this parameter for Alpha from its prevalence in wastewater separately for Lausanne and Zurich on the basis of a logistic progression model (Methods and Extended Data Fig. 2). Our estimates of the transmission fitness advantage of 46% (confidence interval (CI) of 35–60%) for the Zurich WWTP catchment and 59% (CI 42–84%) for the Lausanne WWTP catchment are in line with those based on regional clinical data^19^ and with reports from the United Kingdom^20^ (Supplementary Information). Narrowing the clinical data down to the cantonal level, the estimates are still in line with the wastewater for Zurich (54%, CI 43–69%, based on 2,062 samples), while for Vaud, the clinical estimates are less precise (75%, CI 34–144%, based on 345 samples) as reflected by the huge confidence interval. To assess how early the transmission fitness advantage can be estimated with acceptable precision, we also computed online estimates of the transmission fitness advantage, that is, using only the data up to the respective time point—46 wastewater samples at most per location (Methods). For canton Vaud, the wastewater-based estimates for the Lausanne WWTP are more precise than the estimates based on hundreds of clinical samples from the canton (Fig. 3b). For canton Zurich, the estimates are similar to those of thousands of cantonal clinical samples with one outlier around mid-January for Zurich (Fig. 4b and Supplementary Information). Restricting the clinical data to the 115 samples from the city of Zurich, which comprises the majority of the catchment area of the WWTP, shows that the precision of the wastewater-based online estimates is clearly superior (Fig. 4b and Supplementary Information).

### Early detection of the Delta variant

**Fig. 5:**
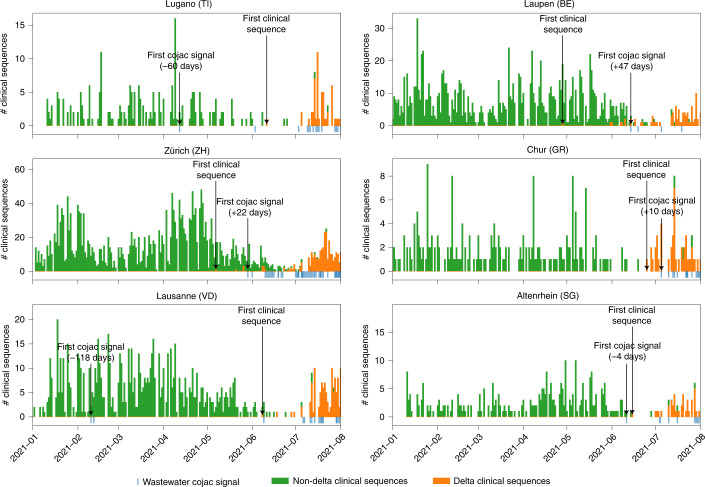
Detection of the Delta variant in six Swiss WWTPs. Detection of the Delta variant in wastewater between January and September 2021 for the WWTPs of Lugano (top left), Laupen (top right), Zürich (middle left), Chur (middle right) and Altenrhein (bottom right), and between January and August 2021 for the WWTP of Lausanne (bottom left). Detection was performed through co-occurrences of signature mutations using COJAC, and compared to clinical sequencing in the cantons where the treatment plants are located. Green and orange bars represent the number of non-Delta and Delta clinical sequences, respectively, from the canton (stacked) for each day in the surveyed period. Blue bars indicate COJAC signals of variant-specific mutation co-occurrences in the wastewater. First detections are indicated by black arrows. Source data

To investigate whether our results could be reproduced for the emergence of the Delta variant (B.1.617.2), we analysed additional data from six WWTPs across Switzerland during the introduction and spread of B.1.617 and all its sublineages (denoted B.1.617*) (Fig. 5) for co-occurring signature mutations. Although the RNA concentration in sewage samples at this time was very low due to a lull in the pandemic, we were able to detect signals of B.1.617*- and B.1.617.2-specific co-occurrences before or early during the local spread of the variant as observed in clinical samples. In three out of six catchment areas, the wastewater-derived signal was detected before confirmation of the first local B.1.617*-positive clinical sample: 118 d earlier in Lausanne, 60 d earlier in Lugano and 4 d earlier in Altenrhein. For the other WWTPs, the variant was first found in the clinical samples of the canton: 10 d earlier in Chur, 22 d earlier in Zurich and 47 d earlier in Laupen. In two of these cases (Lausanne and Lugano), the first detections of the Delta variant were transient and resumed at later dates when the spread of the variant started. In the cases where the detection in wastewater did not precede the detection in clinical samples, the variant was still detected when its prevalence was low. Whether wastewater-based variant detection can precede detection in clinical samples or not depends on the rate at which clinical samples are sequenced. To investigate this effect further, we subsampled the clinical sequences at different sample sizes (Supplementary Fig. 4). We found that in general, for low clinical sample sizes, wastewater-based detection precedes clinical detection, while increasing clinical sample size eventually decreases or reverses the advantage of wastewater, with strong diminishing returns.

## Discussion

We have demonstrated how genomic sequencing of wastewater samples can be used to detect, monitor and evaluate genetic variants of SARS-CoV-2 on a population level. Specifically, we have reported the detection of the local outbreak of the Alpha variant in wastewater in two Swiss cities before it was observed in clinical samples. We expanded our surveillance to six Swiss cities and found that in three of them, the earliest signal of the Delta variant in wastewater pre-dated the first local detection of the variant in clinical samples despite very high clinical sequencing rates at that time in Switzerland (between 66% and 94% of Swiss qPCR-positive samples were randomly selected for sequencing at that time). In the cases where clinical samples provided the first local evidence for the presence of the Delta variant, the first signal in wastewater occurred shortly after and at a time when the local prevalence of the Delta variant was still very low. By subsampling the available clinical samples, we have shown the strong association between the rate of clinical sequencing and the delay in variant detection compared with wastewater-based analyses.

While we have shown that early variant detection based on wastewater samples is feasible, we have also observed that the interpretation of single wastewater samples can be challenging. This is because initially, only a small subset of signature mutations is typically observed at low frequencies, which makes it difficult to distinguish between signal and noise in the sequencing data. The high noise level in the data is attributable to the technical challenges that arise in the collection and processing of the raw wastewater sample, the extraction of SARS-CoV-2 RNA and its amplification. Our results suggest that high sampling density across time and replicate sequencing are key elements to improve the signal-to-noise ratio.

We also developed an approach to boost the signal strength in individual samples. This approach is based on the detection of co-occurring signature mutations on the same read pair, as their presence in a sample constitutes a much stronger signal than individual mutations. This approach was particularly valuable for the detection of the Alpha variant which has multiple highly specific mutation pairs and even one triplet that can be detected in this manner. In Lausanne, the first co-occurrence-based evidence of the Alpha variant occurred 13 d before the first clinical evidence at that time in the area and 8 d earlier than in the clinical samples analysed retrospectively. Among the other variants we studied, Beta and Gamma shared one pair of co-occurring signature mutations, with Gamma having one additional variant-specific mutation pair. We detected some early evidence of the introduction of these variants into the Swiss population, but neither of these two variants was able to establish itself in Switzerland against the Alpha variant. For the Delta variant, there are two pairs of signature mutations, which we used for early co-occurrence-based detection: one shared among B.1.617* and one exclusive to B.1.617.2. In general, the usefulness of the co-occurrence analysis for variant identification depends on the exclusivity of co-occurrences and is negatively affected by the presence of recurring mutations in separate lineages^21,22^. As more and more variants arise and possibly co-exist in a population, shared signature mutations—by chance, convergence or homology—are more likely to occur. In such a case, deconvolution methods^23^ will become useful to disentangle the aggregate signals of co-occurring variants in wastewater. While we focused in this paper on the introduction of known variants into a new population, the data we generated and the methods we developed can in principle be used and extended to a de novo identification of circulating variants and the detection of cryptic variants in unsampled human or non-human animal populations.

Besides early detection, we have shown that sequencing data obtained from wastewater samples can also be used to monitor the local prevalence of a variant, and to estimate its growth rate and transmission fitness advantage earlier and on the basis of substantially fewer samples as compared with using clinical samples. Moreover, wastewater samples have the advantage of representation of undiagnosed asymptomatic cases in the data, which are systematically overlooked in clinical sequencing.

Several challenges remain in the analysis and interpretation of wastewater-derived sequencing data. For example, relating the wastewater-derived estimates to a specific local population can be confounded by the movement of people. Switzerland, as many other European countries, has a high level of commuting between geographical locations within the country and from neighbouring regions. The high congruence we observe between clinical and wastewater samples in terms of estimated prevalence and fitness advantage suggests that there is little difference between these two sources of information in regard to the overall presence of infectious individuals regularly present in a city due to residency or daily commute. Another challenge is posed by potential differences in shedding profiles between variants, which may impact quantification from wastewater sequencing, and thus also impact some of the inferred epidemiological characteristics of the variants.

Overall, we have shown that genomic analysis of SARS-CoV-2 variants in wastewater samples can inform epidemiological studies and complement established approaches based on clinical samples. In fact, we have expanded our continued sequencing activities to six wastewater treatment plants across Switzerland for which we publish live updates for the general public as well as for local public health agencies (https://bsse.ethz.ch/cbg/research/computational-virology/sarscov2-variants-wastewater-surveillance.html). Our methodology and continued sequencing campaign provide a blueprint for rapid, unbiased and cost-efficient genomic surveillance of emerging SARS-CoV-2 variants based on longitudinal sequencing of wastewater samples.

## Methods

### Wastewater sample collection and preparation

**Extended Data Fig. 1:**
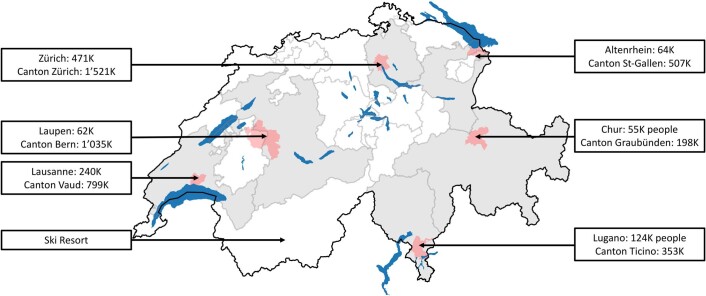
Geographical locations of wastewater treatment plants (WWTPs) surveyed for this study. Geographical locations of wastewater treatment plants (WWTPs) surveyed for this study. WWTP catchment areas are highlighted in light red. Cantons in which the WWTPs are located are highlighted in grey. Population numbers are for the WWTP catchment areas and surrounding cantons, respectively. Location of the ski resort is illustrative. Source: Federal Office of Topography. Wastewater treatment plant catchments of Switzerland: Eawag (2014) updated from https://www.dora.lib4ri.ch/eawag/islandora/object/eawag%3A5599.

**Extended Data Fig. 2:**
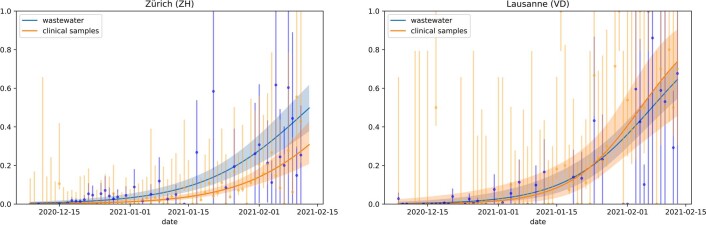
Logistic growth model fitted to variant proportions derived from wastewater and clinical samples. Dots with error bars represent daily empirical proportions of Alpha-positive clinical samples (orange), or average empirical proportions of Alpha-characteristic substitutions in wastewater (blue). Error bars are 95% Wilson confidence intervals. Solid lines represent maximum likelihood fitted values of the logistic model used to infer transmission advantage. Shaded areas represent 95% confidence bands. Source data

Raw wastewater samples were collected from three Swiss WWTPs: Werdhölzli, Zurich (64 samples, July 2020–February 2021, population connected: 450,000), Vidy, Lausanne (49 samples, September 2020–February 2021, population connected: 240,000), and an alpine ski resort (8 samples, December 2020) (Fig. 1a and Extended Data Fig. 1). For the validation study, we used mostly daily wastewater samples from Lugano (*n* = 238, February–September 2021), Laupen (*n* = 242, February–September 2021), Zurich (*n* = 251, January–September 2021), Chur (*n* = 230, February–September 2021), Lausanne (*n* = 186, January–July 2021) and Altenrhein (*n* = 236, February–September 2021). Composite samples (24 h; Zurich and Lausanne) or grab samples (ski resort) were collected in 500 ml polystyrene or polypropylene plastic bottles, shipped on ice and stored at 4 °C for up to 8 d before processing. Aliquots of 50 ml were clarified by filtration (2 µm glass fibre filter (Millipore) followed by a 0.22 µm filter (Millipore), Zurich samples) or by centrifugation (4,863 × *g* for 30 min, Lausanne and ski resort samples). Clarified samples were then concentrated using centrifugal filter units (Centricon Plus-70 Ultrafilter, 10 kDa, Millipore) by centrifugation at 3,000 × *g* for 30 min. Centricon cups were inverted and the concentrate was collected by centrifugation at 1,000 × *g* for 3 min. The resulting concentrate (up to 280 µl) was extracted using the QiaAmp viral RNA mini kit (Qiagen) according to the manufacturer’s instructions, adapted to the larger volumes and eluted in 80 µl. Samples collected after 1 February were further purified using One-Step PCR Inhibitor Removal columns (Zymo Research). RNA extracts were stored at −80 °C for up to 4 months before sequencing.

### Genomic sequencing

RNA extracts from wastewater samples were used to produce amplicons and to prepare libraries according to the COVID-19 ARTIC v3 protocol^24^ with minor modifications. Briefly, extracted RNA was reverse transcribed using the NEB LunaScript RT SuperMix kit (New England Biolabs) and the resulting complementary DNA was amplified with the ARTIC v3 panel from IDT. ARTIC primers used were: ARTIC V4.1 NCOV-2019 Panel, 500rxn 10011442, IDT ARTIC v3 panel 500rxns 10006788 (IDT). The amplicons were end-repaired and polyadenylated before ligation of adapters using NEB Ultra II (New England Biolabs). Fragments containing adapters on both ends were selectively enriched and barcoded with unique dual indexing with PCR. Libraries were sequenced using the Illumina NovaSeq 6000 and MiSeq platforms, resulting in paired-end reads of 250 bp length each (see Supplementary Information for quality metrics of the sequencing data).

### Mutation calling

NGS data were analysed using V-pipe^25^, a bioinformatics pipeline for end-to-end analysis of viral sequencing reads obtained from mixed samples. Individual low-frequency mutations were called on the basis of local haplotype reconstruction using ShoRAH^26^. For detecting mutation co-occurrence, we developed a computational tool called COJAC. The ARTIC v3 protocol relies on tiled amplification and some amplicons cover multiple positions mutated in a variant (Supplementary Table 1). As the samples are sequenced with paired-end 250 bp reads, each 400 bp amplicon can be fully observed on the read pairs in close to all instances. Detecting multiple signature mutations on the same amplicon increases the confidence of mutation calls at very low variant read counts. This opens the possibility of earlier detection while variant concentrations are still too low for reliable detection of individual mutations. COJAC takes as input the multiple read alignments (BAM files) and counts read pairs with variant-specific mutational patterns. It can be configured to work with any tiled amplification scheme and to simultaneously search for multiple variants, each defined by a list of signature mutations. COJAC and its documentation (README file) are available at https://github.com/cbg-ethz/cojac/ or as a bioconda package at https://bioconda.github.io/recipes/cojac/README.html.

### Statistical data analysis

For Zurich, we used the 55 sequencing experiments (excluding 1 failed) covering 46 dates ranging from 8 December 2020 to 11 February 2021. For Lausanne, we used the 52 sequencing experiments (excluding 4 failed) covering 43 dates ranging from 8 December 2020 to 13 February 2021. When a WWTP sample was sequenced multiple times, we fixed the empirical frequencies of the Alpha signature mutations for a given day by averaging their values between the different sequencing experiments. We only used non-synonymous substitutions for quantification. Frequencies of the Alpha signature substitutions in wastewater-derived NGS data were resampled with replacement and averaged per wastewater sample, before being smoothed across time by local regression using locally weighted scatterplot smoothing (lowess) with 1/3 bandwidth from the Python v3.7.7 library statsmodels v0.12.1^27^. This process was repeated 1,000 times to construct bootstrap estimates of the Alpha per-day frequency curves. The smoothed resampled values were used to compute point estimates by averaging the daily Alpha prevalence as well as confidence intervals as the empirical 2.5% and 97.5% quantiles. For the prevalence estimation of Alpha in clinical samples, we used the whole-genome sequencing data from randomly selected SARS-CoV-2 RT-qPCR-positive samples provided by the large diagnostics company Viollier AG^19^. Daily cantonal relative abundances of variants were estimated as their empirical frequencies in sequenced samples. For each canton, the sequenced cases were resampled with replacement and aggregated into daily relative frequencies of Alpha, which were then smoothed temporally using the same lowess smoother mentioned above. This process was repeated 1,000 times to construct bootstrap estimates of the Alpha daily cantonal relative prevalence, which were aggregated into point estimates and confidence intervals by the same method described above.

### Estimation of epidemiological parameters

Following Chen et al.^19^, we assumed that the relative frequency *p*(*t*) of the Alpha variant in the population at time *t* follows a logistic growth with rate *a* and inflection point *t*_0_,

$$p(t) = \frac{{\mathrm{exp}\{ a(t - t_0)\} }}{{1 + \mathrm{exp}\{ a(t - t_0)\} }}.$$

For the wastewater samples, we further assumed that the Alpha signature mutation counts are distributed according to a binomial distribution, with expected value equal to *p*(*t*) times the total coverage at the respective site. Similarly, we assumed that the Alpha-positive clinical samples are also distributed according to a binomial distribution, with expected value equal to *p*(*t*) times the number of clinical samples analysed. The R v3.6.1 package stats^28^ was used to produce maximum likelihood estimates of the model parameters with a generalized linear model. Confidence intervals were computed on the basis of their asymptotically normal distribution. To account for overdispersion due to the inherently noisy nature of wastewater sequencing data, the confidence intervals were computed using the variance of a quasibinomial^29^ distribution. Although clinical data are not expected to exhibit overdispersion, the same procedure was applied for the sake of consistency. Confidence bands were first generated for the linear predictors, and then back-transformed into confidence bands for the regression curves to ensure that they are restricted to the interval (0,1). Estimates of the logistic growth parameter *a* were then transformed into estimates of the transmission fitness advantage *f*_d_, assuming the discrete-time model of Chen et al.^19^ with generation time *g* = 4.8 d such that $f_d = \mathrm{exp}\left( {ag} \right) - 1$. Confidence intervals for the logistic growth parameter *a* were then back-transformed into confidence intervals for the fitness advantage *f*_d_. This inference procedure was repeated at multiple timepoints with only the clinical and wastewater sequencing data available at these timepoints, to generate online estimates and confidence intervals of what could have been inferred about *f*_d_ at that time. These estimates were compared to the estimates of *f*_d_ reported in Chen et al.^19^ for the Lake Geneva region (population 1.6 million), which includes Lausanne and the Greater Zurich Area (population 1.5 million). The confidence intervals for these regional estimates of *f*_d_ were recomputed using back-transformation of the confidence intervals reported for the regional estimates of *a*, so that they could be meaningfully compared with the ones based on our data.

### Dilution experiment

RNA samples of cultivated wild-type SARS-CoV-2 (Wuhan strain) and of a clinical Alpha strain were obtained. We measured the RNA concentrations in these samples by quantifying the N1 gene target (on the basis of the CDC N1 gene assay; primers in Supplementary Table 6) using Crystal Digital PCR (Naica system, Stilla Technologies)^30^. A 27 μl pre-reaction volume for Sapphire Chips (CN C14012, Stilla Technologies) was prepared consisting of 5.4 μl of template, 13.5 μl of 2× qScript XLT One-Step RT-PCR, and N1 primers and probe (2019-nCov RUO kit, CN 100006713, Integrated DNA Technologies). Droplet production and PCR were performed on the Naica Geode. Reverse transcription (48 °C for 50 min) was followed by denaturation (94 °C for 3 min) and 40 cycles (94 °C for 30 s, 57 °C for 1 min) of denaturation and annealing/extension. The Naica Prism3 using the Crystal Reader and Crystal Miner software were used for analysis (Stilla Technologies).

On the basis of the dPCR measurements, each RNA sample was then diluted in an RNA extract produced from SARS-CoV-2-free wastewater (November 2019, Lausanne) to a final concentration of 200 gc µl^−1^. Wild-type and Alpha solutions were then mixed at wild-type to variant ratios of 10:1, 50:1 and 100:1, and each mixture was sequenced 5 times.

### Replicate experiment

RNA extracts of 25 samples taken in the Lausanne and Zurich WWTPs between 8 December 2020 and 4 January 2021 were processed and sequenced a second time. For 23 RNA extracts, sequencing data were successfully produced for both experiments. Another replicate experiment was performed, for which RNA extract was produced as described above from two samples obtained from the Lausanne WWTP on 7 January 2020. The extracts were pooled and subsequently divided into 9 replicate samples for sequencing.

### Patient sequences

Per-patient SARS-CoV-2 consensus sequences were downloaded from GISAID^31^ for all samples collected in Switzerland between 24 February 2020 and 13 February 2021, and not identified as either Alpha, Gamma or Beta (see Supplementary Information for the list of accession numbers).

### Validation experiment for the Delta variant

We used COJAC as described above to call characteristic mutations co-occurring on amplicons 76 (22917G and 22995A, characteristic of B.1.617*) and 91 (27638C and 27752T, characteristic of B.1.617.2) to detect the Delta variant. For each treatment plant, we compared the wastewater sequencing results to clinical consensus sequences of the respective canton for the time period between January and October 2021, which we downloaded from GISAID^31^ through the LAPIS API of Cov-Spectrum^32^. We restricted the clinical sequencing data to samples from the Viollier (AG) laboratory (by selecting sequences where ‘originatingLab‘=‘Viollier AG‘)^19^.

### Reporting summary

Further information on research design is available in the Nature Research Reporting Summary linked to this article.

## Supplementary information

Supplementary Information Supplementary Figs. 1–4, Details and Tables 1–7.

Reporting Summary

Supplementary Data 1 Complete COJAC output for the wastewater samples analysed in this study.

Supplementary Data 2 Detailed acknowledgements of the originating and submitting laboratories of the GISAID data.

## Data Availability

Wastewater sequencing data (depleted from human-derived reads) are available on the European Nucleotide Archive (ENA) under project accession number PRJEB44932. Source data are provided with this paper.

## Source data

Source Data Fig. 1 Statistical source data.
Source Data Fig. 2 Statistical source data.
Source Data Fig. 3 Statistical source data.
Source Data Fig. 4 Statistical source data.
Source Data Fig. 5 Statistical source data.
Source Data Extended Data Fig. 2 Statistical source data.

## Code availability

The code for the automated data analysis is available at https://github.com/cbg-ethz/pangolin. The bioinformatics pipeline V-pipe for the analysis of viral sequencing data is available at https://github.com/cbg-ethz/V-pipe/. The program COJAC for detecting mutation co-occurrence is available at https://github.com/cbg-ethz/cojac/ and also as a package in bioconda at https://bioconda.github.io/recipes/cojac/README.html. The R and Python notebooks used in this Article are available at https://github.com/cbg-ethz/cojac/tree/master/notebooks. Updated version of the notebooks and detailed description are available at https://github.com/cbg-ethz/cowwid.
