# Supplementary material for: Early detection and surveillance of SARS-CoV-2 genomic variants in wastewater using COJAC
Source: Nat Microbiol. 2022 Jul 18;7(8):1151–60. doi: 10.1038/s41564-022-01185-x (PMC9352586; doi:10.1038/s41564-022-01185-x)
Supplement: Supplementary file 1 — Supplementary Figs. 1–4, Details and Tables 1–7. [file 41564_2022_1185_MOESM1_ESM.pdf]

nature portfolio

https://doi.org/10.1038/s41564-022-01185-x

Supplementary information

Early detection and surveillance of SARS-CoV-2 genomic variants in wastewater using COJAC

In the format provided by the authors and unedited

Supplementary information for “Early detection and surveillance of SARS-CoV-2 genomic variants in wastewater using COJAC”

Overview of signature mutations of Alpha, Beta, and Gamma variants

Supplementary Table S1. Signature mutations of the Alpha, Beta, and Gamma lineages, their amplicon location and frequencies among all 21,163 non-Alpha, non-Beta, and non-Gamma consensus sequences available in GISAID and obtained from clinical samples in Switzerland until February 13, 2021.

	Nucleotide			Variant				GISAID	
**Gene**	**position**	**change**	**Amino acid change**	**Alpha**	**Beta**	**Gamma**	**Ampli-cons**	**counts**	**relative freq. (%)**
**ORF1ab**	**733**	T>C	syn	0	0	1	[3]	0	0
**ORF1ab**	**913**	C>T	syn	1	0	0	[3]	20	0.1
**ORF1ab**	**1059**	C>T	T265I	0	1	0	[4]	324	1.53
**ORF1ab**	**2749**	C>T	syn	0	0	1	[9]	0	0.00
**ORF1ab**	**3267**	C>T	T1001I	1	0	0	[11]	48	0.22
**ORF1ab**	**3828**	C>T	S1188L	0	0	1	[13]	5	0.02
**ORF1ab**	**5230**	G>T	K1655N	0	1	0	[17]	77	0.36
**ORF1ab**	**5388**	C>A	A1708D	1	0	0	[18]	35	0.16
**ORF1ab**	**5648**	A>C	K1795Q	0	0	1	[19]	1	0.00
**ORF1ab**	**5986**	C>T	syn	1	0	0	[20]	57	0.27
**ORF1ab**	**6954**	T>C	I2230T	1	0	0	[23]	28	0.13
**ORF1ab**	**10323**	A>G	K3353R	0	1	0	[34]	261	1.23
**ORF1ab**	**11288**	---------	3675-3677 -SGF	1	1	1	[37]	150	0.71
**ORF1ab**	**12778**	C>T	syn	0	0	1	[42, 43]	2	0.01
**ORF1ab**	**13860**	C>T	syn	0	0	1	[46]	66	0.31
**ORF1ab**	**14676**	C>T	syn	1	0	0	[49]	32	0.15
**ORF1ab**	**15279**	C>T	syn	1	0	0	[51]	62	0.29
**ORF1ab**	**16176**	T>C	syn	1	0	0	[53, 54]	29	0.14
**S**	**21621**	C>A	T20N	0	0	1	[71]	0	0
**S**	**21638**	C>T	P26S	0	0	1	[71]	38	0.18
**S**	**21765**	------	69-70 -HV	1	0	0	[72]	1054	4.98
**S**	**21801**	A>C	D80A	0	1	0	[72]	0	0.00
**S**	**21991**	---	144 -Y	1	0	0	[72, 73]	68	0.32
**S**	**22812**	A>C	K417T	0	0	1	[75]	0	0

**S**	**22813**	G>T	K417N	0	1	0	[75]	0	0.00
**S**	**23012**	G>A	E484K	0	1	1	[76]	43	0.20
**S**	**23063**	A>T	N501Y	1	1	1	[76]	68	0.32
**S**	**23271**	C>A	A570D	1	0	0	[77]	25	0.12
**S**	**23604**	C>A	P681H	1	0	0	[78]	243	1.15
**S**	**23664**	G>T	A701V	0	1	0	[78]	4	0.02
**S**	**23709**	C>T	T716I	1	0	0	[78]	123	0.58
**S**	**24506**	T>G	S982A	1	0	0	[81]	20	0.09
**S**	**24642**	C>T	T1027I	0	0	1	[81]	24	0.11
**S**	**24914**	G>C	D1118H	1	0	0	[82]	27	0.13
**ORF3a**	**25563**	G>T	Q57H	0	1	0	[84]	6532	30.87
**ORF3a**	**25904**	C>T	S171L	0	1	0	[85]	49	0.23
**E**	**26456**	C>T	P71L	0	1	0	[87]	16	0.08
**ORF8**	**27972**	C>T	Q27stop	1	0	0	[92]	62	0.29
**ORF8**	**28048**	G>T	R52I	1	0	0	[92]	62	0.29
**ORF8**	**28111**	A>G	Y73C	1	0	0	[92, 93]	23	0.11
**ORF8**	**28167**	G>A	E92K	0	0	1	[93]	7	0.03
**N**	**28280**	GAT>CTA	D3L	1	0	0	[93]	29	0.14
**N**	**28512**	C>G	P80R	0	0	1	[94]	0	0
**N**	**28887**	C>T	T205I	0	1	0	[95]	178	0.84
**N**	**28977**	C>T	S235F	1	0	0	[95]	42	0.2

Quality of NGS data

DNA libraries were prepared from the viral RNA extracted from the wastewater samples using the ARTIC V3 protocol with minor modifications. Briefly, extracted RNA was reversed transcribed using the NEB LunaScript RT SuperMix Kit (E3010L) and the resulting cDNA was amplified with the ARTIC v3 panel from IDT(10006788). The amplicons were end-repaired and polyadenylated before ligation of adapters using NEB Ultra II (E7645L). Fragments containing adapters on both ends were selectively enriched and barcoded with unique dual indexing with PCR. Libraries were sequenced using 2 x 250bp paired-end reads (Methods). After quality control, we obtained a median number of reads per sample of 949,884. In the subsequent read mapping step, we successfully aligned a median of 472,278 reads per sample, which resulted in a median per-sample median coverage of 1539 reads per position of the SARS-CoV-2 genome (range 0 - 28,958). The median number of positions with insufficient coverage (<5 reads) was 910 positions, and the median number of positions with low coverage (<50 reads) was 1702 positions. As a reference, over the same period of time, the clinical swab test samples yielded a median number of reads per sample of 2,400,100, and we successfully aligned a median of 1,012,662 reads per clinical sample, which resulted in a median per-sample median coverage of 2832 reads per position of the SARS-CoV-2 genome.

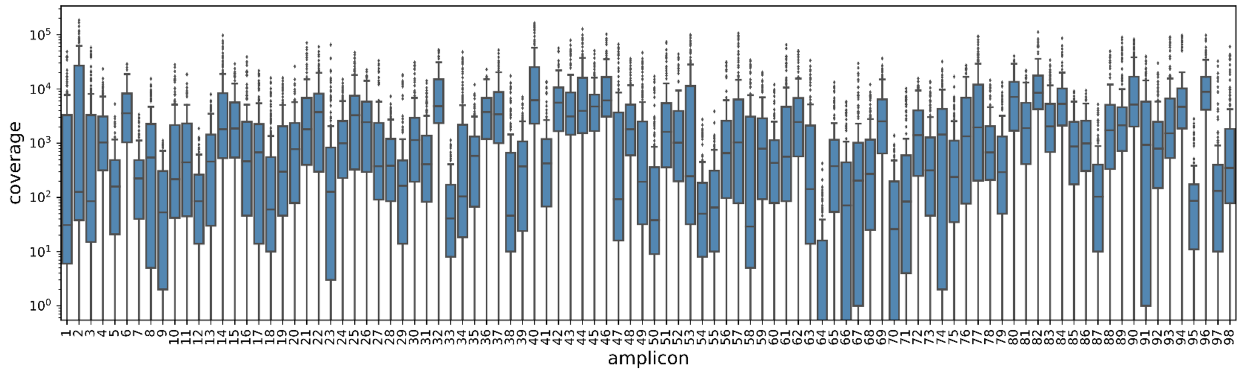

Supplementary Figure S1. Distributions of per-amplicon coverage in all 121 wastewater samples after quality filtering and alignment. Boxes show quartiles and the whiskers extend to a maximum of 1.5 times the inter quartile range, after which points are considered outliers.

Reproducibility of variant detection and quantification

We selected 24 samples (Supplementary Table 2) for resequencing in a separate sequencing run and compared sample-specific fraction of reads supporting Alpha signature mutations averaged over all signature mutations (Figure 1B). Most of the resequenced samples originate from the second half of December 2020 to early January 2021, the time frame in which the Alpha variant emerged in the Swiss population. We observe a low correlation between the fraction pairs (Pearson correlation coefficient R^2 = 0.35, p < 0.11, two-sided test) which indicates a high degree of variability of Alpha prevalence estimates at low frequencies.

To assess the influence of variant concentration on sensitivity, we sequenced pre-pandemic wastewater samples spiked with mixes of SARS-CoV-2 RNA at three different Alpha-to-wild-type ratios, 10:1, 50:1 and 100:1 (Methods). Each mixture was sequenced five times (Fig 1D). Overall, the estimated fraction of Alpha displayed high correlation between replicates: in an analysis of variance (ANOVA) dilution ratios accounted for 44.9% ($F=120.87$, $df=2$, $p< 2.2e-16$) of the variance in Alpha mutation frequencies, with higher relative precision at higher concentration (Supplementary Figure S2). Mutation positions accounted for 19.83% ($F=7.12$, $df=15$, $p=2.851e-12$) of the variance in Alpha mutation frequencies (while controlling for dilution), with some mutation frequencies consistently underestimated or overestimated, possibly due to background prevalence of mutations or effects of mutations on primer annealing.

Supplementary Table S2. List of 25 samples resequenced to assess reproducibility of Alpha prevalence estimates (Fig. 1B). Samples have been sequenced two times in total, except for the sample taken in Lausanne on October 15 which was sequenced three times.

Sample	Date	Location
C6_12_2021_01_04	2021-01-04	Lausanne (VD)
G2_12_2021_01_02	2021-01-02	Lausanne (VD)
F2_12_2020_12_31	2020-12-31	Lausanne (VD)
E2_12_2020_12_27	2020-12-27	Lausanne (VD)
D2_12_2020_12_25	2020-12-25	Lausanne (VD)
C2_12_2020_12_23	2020-12-23	Lausanne (VD)
B2_12_2020_12_19	2020-12-19	Lausanne (VD)
G1_12_2020_12_17	2020-12-17	Lausanne (VD)
A2_12_2020_12_15	2020-12-15	Lausanne (VD)
F1_12_2020_12_13	2020-12-13	Lausanne (VD)
E1_12_2020_12_10	2020-12-10	Lausanne (VD)
D1_12_2020_12_09	2020-12-09	Lausanne (VD)
C1_12_2020_12_08	2020-12-08	Lausanne (VD)
A1_12_2020_10_15	2020-10-15	Lausanne (VD)
B1_12_2020_10_15_n	2020-10-15	Lausanne (VD)
D4_10_2021_01_04	2021-01-04	Zurich (ZH)
C4_10_2021_01_02	2021-01-02	Zurich (ZH)
B4_10_2020_12_31	2020-12-31	Zurich (ZH)
A4_10_2020_12_29	2020-12-29	Zurich (ZH)
H3_10_2020_12_25	2020-12-25	Zurich (ZH)
G3_10_2020_12_23	2020-12-23	Zurich (ZH)
F3_10_2020_12_21	2020-12-21	Zurich (ZH)
H1_10_2020_12_20	2020-12-20	Zurich (ZH)
E3_10_2020_12_19	2020-12-19	Zurich (ZH)
D3_10_2020_12_17	2020-12-17	Zurich (ZH)

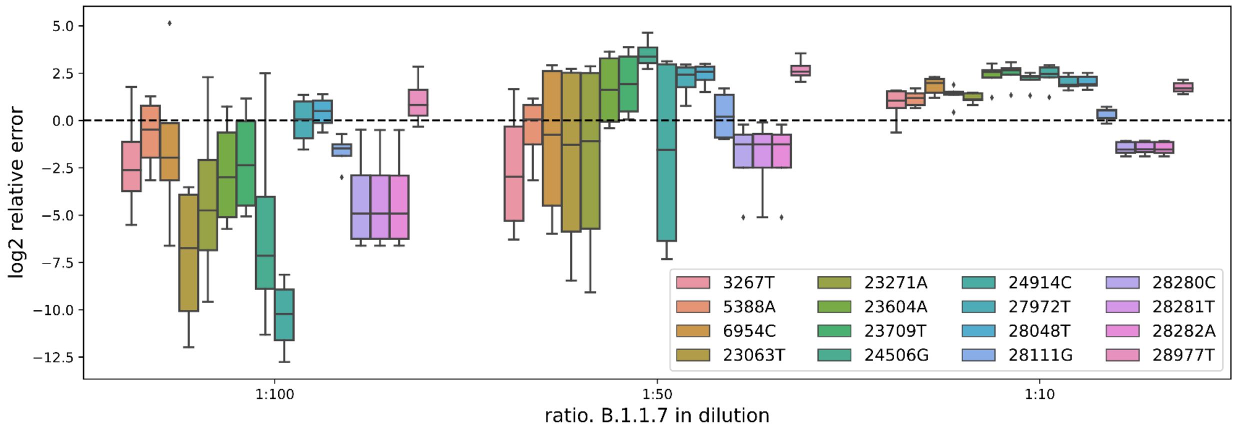

Supplementary Figure S2. Estimation error in dilution experiments. Log2 of relative overestimation/underestimation of the fraction of Alpha (B.1.1.7) characteristic substitutions are reported, i.e. Log2 of proportion of mutation in sequencing data minus log2 of the proportion of Alpha RNA in dilution, in five technical replicates of wastewater spiked with SARS-CoV-2 RNA at 3 different Alpha-to-wild-type ratios.. Boxes show quartiles and the whiskers extend to a maximum of 1.5 times the inter quartile range, after which points are considered outliers.

To further assess reproducibility, RNA extracts from two samples from the Lausanne WWTP of January 7, 2021 were pooled and subsequently divided into 9 replicate samples for sequencing. Alpha fractions were estimated independently for each replicate. Estimates ranged from 0 to 0.1, had an average of 0.04 and a variance of 0.002 (Supplementary Figure S3), while the estimate of the prevalence of Alpha from temporally smoothed Lausanne WWTP samples was 0.07 (Figure 4). In the seven replicate samples with evidence of Alpha, we detected a median of five out of 16 Alpha characteristic nonsynonymous substitutions.

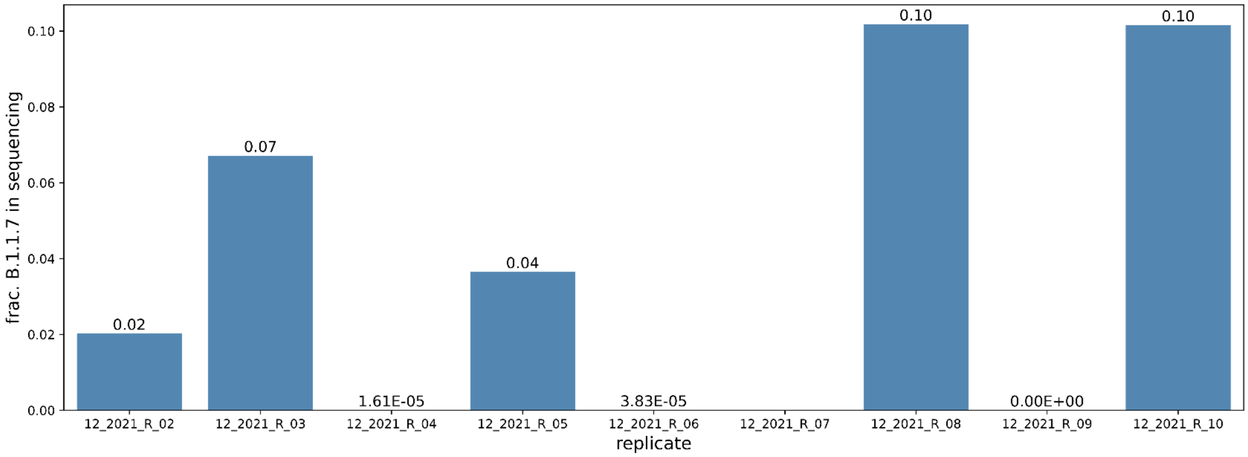

Supplementary Figure S3. Estimates of the fraction of Alpha (B.1.1.7) in the resequencing experiments of the January 7, 2021, Lausanne WWTP sample. Estimate from the smoothed time series is ~0.07.

We take these results to indicate that quantification of the prevalence of a variant is possible, and that replication can increase the precision, especially when monitoring low frequency variants.

Detection of Beta and Gamma variants in the wastewater samples

Unlike for Alpha, we found almost no evidence for the distinctive signature mutations of Beta or Gamma. The frequently observed mutations G25563T (Beta) and A23063T (Beta and Gamma) are non-distinctive, as the former has been observed independently in the Swiss population prior to the emergence of Beta and the latter is shared with the more prevalent Alpha variant. The absence of Beta and Gamma in our data is consistent with the extremely low prevalence of these variants in Switzerland estimated from clinical samples. Between January and February 2021, out of 1338 clinical samples sequenced in Zurich and 466 in Vaud, Gamma was detected only once in Zurich and not at all in Vaud, while Beta was observed two times in Zurich and seven times in Vaud.

Detection of mutation co-occurrence using Cojac

To validate Cojac, we first searched for the characteristic mutational patterns in two Alpha-positive, two Beta-positive, and one Gamma-positive control patient samples and indeed detected co-occurrence of the respective mutations on essentially all reads (Supplementary Table 3, last five rows). In addition, all patient and wastewater samples displayed, as expected, on amplicon 77 the A23403G signature mutation of the B.1 lineage, which is the most prevalent lineage (>99%) in Switzerland at the time of sampling[1,2].

We then analyzed all 122 wastewater samples from the time series in Figure 2 with cojac. Supplementary Table 2 shows the results for a subset of samples. The full cojac output can be found in Supplementary Data 1. For Lausanne, first evidence of the Alpha variant appears on December 9 in the form of the two co-occurring signature mutations on amplicon 93. This is around the same time as we start to detect individual Alpha signature mutations in Lausanne. GISAID based background frequencies of these two mutations are extremely low (Supplementary Table 4). The combination of both mutations has only been observed in 0.07% of the 21,163 Swiss clinical samples that were not classified as Alpha, Beta, or Gamma (until February 13, 2021). This makes it highly unlikely that the observed signal is originating from other sources than Alpha-infected individuals. Single amplicon-based co-occurrence of Alpha signature mutations continues throughout December (Supplementary Data 1). In early January, we start to see direct evidence of co-occurrence in three and later all four amplicons. (Supplementary Table 3, Supplementary Data 1).

Co-occurrence of N501Y and E484K which are shared by Beta and Gamma is first observed on January 21 (amplicon 76). On February 5, all 135 read pairs covering the amplicon exhibit the two mutations, while two days later, we find hardly any evidence of the co-occurrence. This volatility in combination with the fact that a subset of the Alpha strain has also acquired the E484K mutation in addition to the pre-existing N501Y mutation makes this particular co-occurrence a suboptimal marker for Beta and Gamma detection. For Gamma, two mutations occurring on amplicon 95 provide another opportunity for detecting the variant. However, so far only a single sample (February 7) provides evidence of the co-occurrence of these mutations. Looking at background mutation frequencies, we also observe that this mutation pair is not exclusive to Gamma, but has been observed in 47 out of the 21,163 Swiss GISAID samples not classified as Alpha, Beta, or Gamma. Therefore, also the amplicon-based analysis for Lausanne does not provide clear evidence for the spread of Beta or Gamma.

For Zurich, the first weak evidence of the Alpha variant appears on December 17 in the form of the two co-occurring mutations on amplicon 93 which was also the earliest indicator of the variant in Lausanne. As discussed above, these mutations are unlikely to appear due to other sources than Alpha-infected individuals. Like for Lausanne, we later also observe mutation co-occurrence based on the other three Alpha-specific amplicons. Three out of four are observed for the first time on December 22, all four on December 25. The samples from Zurich analyzed in this study did not provide any evidence for amplicon-based co-occurrence of Beta- or Gamma-specific mutations.

Finally, the daily samples of the ski resort for the time interval between December 20 and 29 consistently show amplicon-based co-occurrence of Alpha mutations, with between one and three amplicons carrying the respective combinations of mutations.

Supplementary Table S3. Co-occurrence of signature mutations. For a subset of selected samples, genomic regions containing two or more Alpha, Beta, or Gamma signature mutations observable on individual read pairs were analyzed. The first column indicates the sample date and location, or ID. Columns 2-7 are labeled with amplicons, their respective genomic regions and the signature mutations they contain. Displayed in columns 2-7 are the number of amplicons with all signature mutations present simultaneously, (separated by a slash) the total coverage by single amplicons, and the fraction of co-occurring mutant reads. Column seven reports the signature mutation (D614G) of lineage B.1, as a control. A variant is considered to be present in a sample if at least 5 read pairs and at least 0.1% of the total number of read pairs covering the amplicon carry all signature mutations of that region. The table shows a small subset of the wastewater samples and five patient samples known to derive from the Alpha, Beta, and Gamma lineages as positive controls. Colors indicate observations indicating the presence of Alpha (yellow), Beta or Gamma (red), Gamma (blue) and B.1 (purple). Results from samples that were sequenced twice appear in two separate rows.

Sample	**Amplicon 72 21682-22013**<br><br>**21765-21770Δ, 21991-21993Δ (Alpha)**	**Amplicon 78 23466-23822**<br><br>**C23604A, C23709T (Alpha)**	**Amplicon 92 27809-28144**<br><br>**C27972T, G28048T, A28111G (Alpha)**	**Amplicon 93 28105-28441**<br><br>**A28111G, 28280GAT→CTA (Alpha)**	**Amplicon 76 22822-23188**<br><br>**G23012A, A23063T (Beta, Gamma)**	**Amplicon 95 28699-29041**<br><br>**A28877T, G28878C (Gamma)**	**Amplicon 77 23145-23499**<br><br>**A23403G (D614G)**
2020-12-25<br>Ski-resort	362 / 2729<br>13.26%	0 / 1114<br>0.00%	87 / 892<br>9.75%	39 / 1143<br>3.41%	0 / 2273<br>0.00%	0 / 91<br>0.00%	1379 / 1380<br>99.93%
2020-12-23<br>Ski-resort	257 / 1194<br>21.52%	0 / 368<br>0.00%	25 / 316<br>7.91%	109 / 706<br>15.44%	0 / 920<br>0.00%	0 / 53<br>0.00%	586 / 586<br>100.00%
2020-12-21<br>Ski-resort	0 / 990<br>0.00%	0 / 2367<br>0.00%	514 / 3689<br>13.93%	0 / 20672<br>0.00%	0 / 165<br>0.00%	0 / 788<br>0.00%	36208 / 36209<br>100.00%
2021-02-07<br>Lausanne	6177 / 9289<br>66.50%	2393 / 2394<br>99.96%	2887 / 4449<br>64.89%	7004 / 7664<br>91.39%	1 / 8556<br>0.01%	20 / 435<br>4.60%	6945 / 6947<br>99.97%
2021-02-05<br>Lausanne	0 / 13568<br>0.00%	0 / 0<br>NA	1492 / 4650<br>32.09%	0 / 2211<br>0.00%	135 / 135<br>100.00%	0 / 133<br>0.00%	0 / 0<br>NA
2021-01-21<br>Lausanne	662 / 6356<br>10.42%	306 / 988<br>30.97%	143 / 815<br>17.55%	169 / 711<br>23.77%	31 / 27238<br>0.11%	0 / 4<br>0.00%	586 / 586<br>100.00%
2021-01-12<br>Lausanne	16 / 128<br>12.50%	6 / 30<br>20.00%	2 / 5<br>40.00%	30 / 354<br>8.47%	3 / 26931<br>0.01%	0 / 0<br>NA	69 / 69<br>100.00%
2021-01-04<br>Lausanne	0 / 3112<br>0.00%	105 / 3963<br>2.65%	26 / 999<br>2.60%	47 / 742<br>6.33%	0 / 8232<br>0.00%	0 / 376<br>0.00%	621 / 623<br>99.68%
2021-01-04<br>Lausanne	0 / 2852<br>0.00%	0 / 595<br>0.00%	32 / 360<br>8.89%	49 / 670<br>7.31%	0 / 6909<br>0.00%	0 / 27<br>0.00%	825 / 826<br>99.88%
2020-12-25<br>Lausanne	0 / 524<br>0.00%	0 / 448<br>0.00%	0 / 47<br>0.00%	21 / 219<br>9.59%	0 / 16720<br>0.00%	0 / 150<br>0.00%	213 / 213<br>100.00%
2020-12-21<br>Lausanne	0 / 4<br>0.00%	0 / 21<br>0.00%	0 / 10<br>0.00%	93 / 3393<br>2.74%	0 / 0<br>NA	0 / 2<br>0.00%	10 / 10<br>100.00%

2020-12-17 Lausanne	0 / 460 0.00%	0 / 322 0.00%	5 / 457 1.09%	0 / 40213 0.00%	0 / 76 0.00%	0 / 13 0.00%	12846 / 12847 99.99%
2020-12-09 Lausanne	0 / 2338 0.00%	0 / 1894 0.00%	0 / 295 0.00%	15 / 352 4.26%	0 / 5092 0.00%	0 / 13 0.00%	276 / 276 100.00%
2020-12-29 Zürich	0 / 2776 0.00%	0 / 1152 0.00%	16 / 828 1.93%	18 / 879 2.05%	0 / 4093 0.00%	0 / 97 0.00%	1250 / 1250 100.00%
2020-12-29 Zürich	56 / 1906 2.94%	54 / 1489 3.63%	34 / 645 5.27%	17 / 550 3.09%	0 / 5084 0.00%	0 / 17 0.00%	410 / 410 100.00%
2020-12-25 Zürich	187 / 2689 6.95%	0 / 1387 0.00%	0 / 1331 0.00%	15 / 1070 1.40%	0 / 2303 0.00%	0 / 178 0.00%	1961 / 1961 100.00%
2020-12-25 Zürich	416 / 4034 10.31%	345 / 4009 8.61%	151 / 1627 9.28%	152 / 1431 10.62%	1 / 4270 0.02%	0 / 325 0.00%	712 / 730 97.53%
2020-12-23 Zürich	0 / 2023 0.00%	0 / 953 0.00%	32 / 957 3.34%	1 / 1059 0.09%	0 / 3245 0.00%	0 / 158 0.00%	1815 / 1815 100.00%
2020-12-23 Zürich	0 / 723 0.00%	18 / 414 4.35%	18 / 266 6.77%	4 / 531 0.75%	0 / 1327 0.00%	0 / 89 0.00%	75 / 75 100.00%
2020-12-22 Zürich	149 / 3453 4.32%	0 / 946 0.00%	30 / 1282 2.34%	114 / 1642 6.94%	0 / 5092 0.00%	0 / 160 0.00%	2581 / 2581 100.00%
2020-12-20 Zürich	0 / 6509 0.00%	0 / 10734 0.00%	154 / 13504 1.14%	0 / 82020 0.00%	0 / 802 0.00%	0 / 1141 0.00%	93625 / 93659 99.96%
2020-12-20 Zürich	0 / 652 0.00%	0 / 428 0.00%	5 / 89 5.62%	4 / 295 1.36%	0 / 5925 0.00%	0 / 94 0.00%	142 / 142 100.00%
2020-12-17 Zürich	0 / 5906 0.00%	0 / 9112 0.00%	0 / 9208 0.00%	0 / 38732 0.00%	0 / 233 0.00%	0 / 887 0.00%	34928 / 34939 99.97%
2020-12-17 Zürich	0 / 2459 0.00%	0 / 994 0.00%	0 / 639 0.00%	5 / 859 0.58%	0 / 9224 0.00%	0 / 40 0.00%	600 / 600 100.00%
Clinical Beta sample 410256	0 / 8314 0.00%	0 / 23023 0.00%	0 / 20487 0.00%	0 / 16822 0.00%	156 / 156 100.00%	0 / 23360 0.00%	32633 / 32699 99.80%
Clinical Beta sample 410279	0 / 1354 0.00%	0 / 1401 0.00%	0 / 2601 0.00%	0 / 3526 0.00%	8 / 8 100.00%	0 / 3738 0.00%	6570 / 6574 99.94%
Clinical Alpha sample 420389	212 / 214 99.07%	236 / 236 100.00%	389 / 389 100.00%	1498 / 1501 99.80%	0 / 3 0.00%	0 / 418 0.00%	3184 / 3184 100.00%
Clinical Alpha sample 420394	82 / 82 100.00%	109 / 109 100.00%	207 / 207 100.00%	739 / 742 99.60%	0 / 7 0.00%	0 / 617 0.00%	2067 / 2068 99.95%
Clinical Gamma sample 471206	0 / 2796 0.00%	0 / 3634 0.00%	0 / 47 0.00%	0 / 1028 0.00%	2660 / 2676 99.40%	247 / 259 95.37%	429 / 429 100.00%

Supplementary Table S4. Background frequencies of mutation co-occurrence in genomic regions (amplicons) containing two or more Alpha, Beta, or Gamma signature mutations. Frequencies are based on the 21,163 non-Alpha, non-Beta, and non-Gamma consensus sequences available in GISAID and obtained from clinical samples in Switzerland until February 13, 2021.

Variant	Amplicon	Mutations	Absolute frequency	Relative frequency (%)
Alpha	72	del 21765-21770	1054	4.98
		del 21991-21993	68	0.32
		co-occurrence	16	0.08
	78	C23604A	243	1.15
		C23709T	123	0.58
		co-occurrence	35	0.17
	92	C27972T	62	0.29
		G28048T	62	0.29
		A28111G	23	0.11
		co-occurrence	12	0.06
	93	A28111G	23	0.11
		GAT28280CTA	29	0.14
		co-occurrence	14	0.07
Beta/ Gamma	76	G23012A	43	0.20
		A23063T	68	0.32
		co-occurrence	0	0.00
Gamma	95	A28877T	50	0.24
		G28878C	47	0.22
		co-occurrence	47	0.22
Gamma	77	A23403G	20710	97.86

Estimation of epidemiological parameters

For any emerging variant, it is important to estimate its transmission fitness as early as possible in order to evaluate whether the variant may be of concern or not. To assess whether wastewater-based genomics can inform about fitness of the Alpha variant, we fit a discrete time logistic growth epidemiological model [3] to the increase in Alpha prevalence (Supplementary Figure S5). We estimated a transmission fitness advantage of 46% (CI 35%-60%) for Zurich and 59% (CI 42%-84%) for Lausanne. These estimates are coherent with those based on the regional clinical data reported in Chen et al. [3], namely 52% (40%-70%) for the greater Zurich area (based on 1590 samples dated December 14 2020 to February 11 2021) and 71% (54%-96%) for the Lake Geneva region around Lausanne (based on 548 samples dated December 14 2020 to February 11 2021). They are also in line with the 40%-70% transmission fitness advantage of Alpha that has been reported in the United Kingdom [4]. To examine how early the transmission fitness advantage could be meaningfully estimated, we fit the model multiple times on the portions of data available up to different timepoints, using data from wastewater, cantonal clinical samples, and city clinical data (Figures 3B and 4B). For Zurich, the estimates based on wastewater and cantonal clinical samples appear to follow the same trajectory, with the latter stabilizing about a week earlier. The delay in stabilization of the estimates derived from Zurich WWTP samples in comparison to those based on cantonal clinical data appears to be driven by two outliers in mid-January (Extended Data Figure 2). In contrast, the estimates based on the city-wide sequencing data (of the city having the greatest overlap in population with the population connected to the WWTP) are not informative during this timeframe. For Lausanne, the estimates based on wastewater appear to start converging weeks before those based on cantonal clinical samples. The Lausanne city (having the greatest overlap in population with the population connected to the Vidy WWTP) clinical data did not contain any Alpha-positive sample. Thus, the online fitness estimates based on 46 and 43 pooled wastewater samples tend to be similar and in some cases superior to those obtained from 2062 and 345 individual clinical samples, respectively.

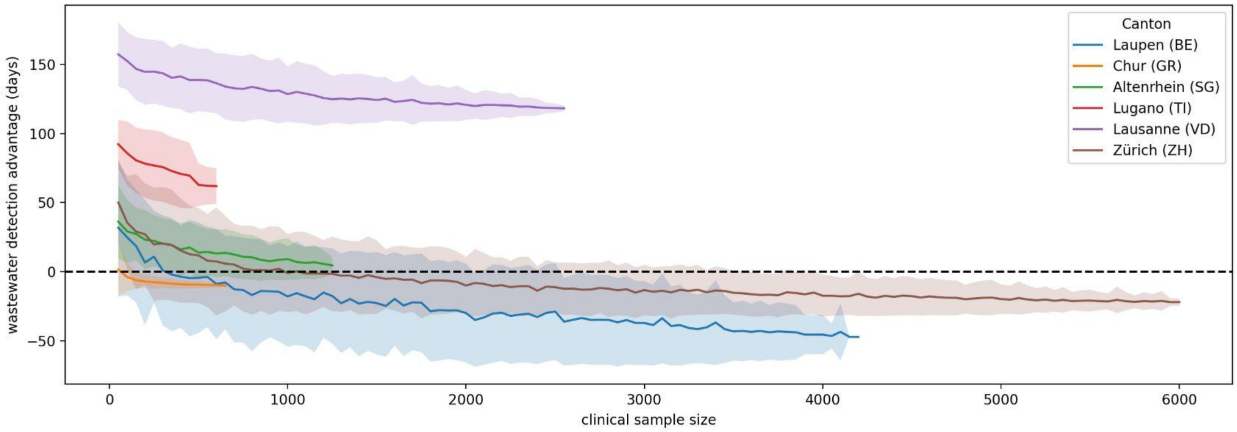

Supplementary Figure S4. Comparison of first detection of the Delta variant in cantonal clinical samples with first detection of the variant in WWTPs. For each cantonal dataset, the SARS-CoV-2 samples have been subsampled 100 times each at varying sample sizes. The figure displays the mean wastewater detection advantage (i.e., the number of days between the first detection in wastewater and the first detection in the subsampled cantonal dataset) as a function of the sample size of the subsampling. Shaded bands are ±1.96 times the resampling standard deviations.

Early Alpha-positive clinical samples

Supplementary Table S5. Overview of all early clinical samples from canton Zurich and canton Vaud that were identified as Alpha (cut-off date December 22, 2020). Gray rows correspond to samples that were only detected in a retrospective sequencing campaign conducted in March/April 2020 and therefore were not available for early detection of the Alpha variant.

Sample	Sample date	Canton	Variant	Originating lab	Submitting lab	Submission date
Switzerland/VD-ETHZ-560572/2020	Dec 17, 2020	Vaud	Alpha	Viollier AG	D-BSSE, ETH Zurich	Apr 22, 2021
Switzerland/VD-UZH-IMV144/2020	Dec 21, 2020	Vaud	Alpha	Dr. Boubaker Karim laboratory	Institute of Medical Virology, University of Zurich	Jan 5, 2021
Switzerland/VD-ETHZ-6/2020	Dec 21, 2020	Vaud	Alpha	MCL Medizinische Laboratorien Hauptstandort Niederwangen	D-BSSE, ETH Zurich	Mar 3, 2021
Switzerland/VD-UZH-IMV143/2020	Dec 22, 2020	Vaud	Alpha	Dr. Boubaker Karim laboratory	Institute of Medical Virology, University of Zurich	Jan 5, 2021
Switzerland/ZH-ETHZ-500087/2020	Nov 09, 2020	Zurich	Alpha	Viollier AG	D-BSSE, ETH Zurich	Mar 2, 2021

Switzerland/ZH-ETHZ-500088/2020	Nov 09, 2020	Zurich	Alpha	Viollier AG	D-BSSE, ETH Zurich	Mar 2, 2021
Switzerland/ZH-ETHZ-500086/2020	Nov 09, 2020	Zurich	Alpha	Viollier AG	D-BSSE, ETH Zurich	Mar 2, 2021
Switzerland/ZH-UZH-IMV130/2020	Dec 18, 2020	Zurich	Alpha	University Hospital Zurich	Institute of Medical Virology, University of Zurich	Dec 30, 2020

Supplementary Table S6. Primers for CDC N1 used for quantification of viral RNA via digital PCR.

Description	Oligonucleotide Sequence (5'>3')	Label
2019-nCoV_N1 Forward Primer	GAC CCC AAA ATC AGC GAA AT	None
2019-nCoV_N1 Reverse Primer	TCT GGT TAC TGC CAG TTG AAT CTG	None
2019-nCoV_N1 Probe	ACC CCG CAT TAC GTT TGG TGG ACC	5' FAM/ZEN 3' IBFQ

Supplementary Table S7. Ids of samples, experiments, and runs stored under the project accession number PRJEB44932[1] on European Nucleotide Archive (ENA), where the viral sequencing raw data has been made available after depletion of human reads.

BioSample	Experiment	Run	Content
SAMEA8745766	ERX5562765 - ERX5562774	ERR5922163 - ERR5922172	Repeated sequencing
SAMEA8745767 - SAMEA8745769	ERX5562775 - ERX5562789	ERR5922173 - ERR5922187	Dilution series<br>The dilution (1:10, 1:50 and 1:100) is indicated in the Sample Title.
SAMEA8745770 - SAMEA8745892	ERX5562790 - ERX5562937	ERR5922188 - ERR5922335	Wastewater samples used for alpha
SAMEA110148731 - SAMEA110150374	ERX9406278 - ERX9407933	ERR9861200 - ERR9862855	Additional wastewater samples used for validation<br>Dates to be filtered as in material and

[1] https://www.ebi.ac.uk/ena/browser/view/PRJEB44932?show=reads

			methods.

Bibliography

1. Alm, E. *et al.* Geographical and temporal distribution of SARS-CoV-2 clades in the WHO European Region, January to June 2020. *Euro Surveill.* **25**, (2020).
2. Stange, M. *et al.* SARS-CoV-2 phylogeny during the early outbreak in the Basel area, Switzerland: import and spread dominated by a single B.1 lineage variant (C15324T). *medRxiv* (2020) doi:10.1101/2020.09.01.20186155.
3. Chen, C. *et al.* Quantification of the spread of SARS-CoV-2 variant B.1.1.7 in Switzerland. *Epidemics* **37**, 100480 (2021).
4. Volz, E. *et al.* Transmission of SARS-CoV-2 Lineage B.1.1.7 in England: Insights from linking epidemiological and genetic data. *medRxiv* (2021) doi:10.1101/2020.12.30.20249034.
